# Systemic Lupus Erythematosus vs. Mixed Connective Tissue Disease Disguised As Scabies

**DOI:** 10.7759/cureus.23621

**Published:** 2022-03-29

**Authors:** Richard Liang, Ian Landry, Merjona Saliaj

**Affiliations:** 1 Medicine, New York Institute of Technology College of Osteopathic Medicine, New York, USA; 2 Medicine, NYC Health + Hospitals/Queens, New York, USA; 3 Medicine, Icahn School of Medicine at Mount Sinai, New York, USA

**Keywords:** lupus scleroderma overlap syndrome, rash, scabies, mixed connective tissue disease, scleroderma, systemic lupus erythematosus

## Abstract

Systemic lupus erythematosus (SLE) is a chronic autoimmune disease that involves numerous body systems and may initially present as a malar or discoid rash. When there are features of other autoimmune disorders present, such as scleroderma or polymyositis, the term mixed connective tissue disorder (MCTD) may be used. The current literature illustrates that the diagnostic criteria for MCTD are unclear. Additionally, the literature presents cases of scabies mimicking the presentation of various autoimmune disorders with cutaneous manifestations. We introduce a case of a young African American female who developed a pruritic rash on her face, abdomen, hands, and scalp. An initial diagnosis of scabies was made, however, failed to improve after a course of permethrin 5% topical cream. Subsequently, she was hospitalized, and diagnosed with pancytopenia, cardiomyopathy, microscopic hematuria and trace proteinuria. Systemic lupus erythematosus/scleroderma overlap syndrome was later confirmed by serologic testing. The patient was started on corticosteroids, with resultant improvement in her symptoms and laboratory findings.

## Introduction

Systemic lupus erythematosus (SLE) is an autoimmune connective tissue disorder predominately affecting women [1]. It has varied clinical manifestations including renal, cardiac, hematologic, integumentary, gastrointestinal, and even neuropsychiatric involvement [2]. The etiology is thought to be secondary to autoreactive B and T cells, along with a loss of immune tolerance against self-antigens [2]. The initial presentation of SLE may mimic a viral syndrome including non-specific symptoms such as weight loss, fatigue, low-grade fever, and arthralgias [2]. A hallmark sign of SLE is the malar (butterfly) rash defined as an erythematous, hyperpigmented, maculopapular rash over the cheeks and nasal bridge that spares the nasolabial folds [3]. This rash is occasionally painful or pruritic and may last from days to weeks [4]. Another cutaneous manifestation of SLE is a discoid rash which appears as well-demarcated, coin-shaped, erythematous scaly plaques of various sizes containing areas of follicular hyperkeratosis [4,5]. There is now a broad range of treatment options for SLE including immunosuppressive therapies, biologic therapies, antimalarial agents and nonsteroidal anti-inflammatory drugs (NSAIDs) but glucocorticoids remain the first-line agents for most SLE manifestations [2]. Treatment advancements and earlier diagnosis of SLE have significantly decreased the mortality rate of this debilitating disease [2].

Another autoimmune condition that shares some of the features of SLE is mixed connective tissue disease (MCTD) [6]. Mixed connective tissue disease is a chronic autoimmune disorder that has characteristics of SLE, systemic sclerosis/scleroderma (SSc), and polymyositis/dermatomyositis (PM/DM) without fulfilling the diagnostic criteria for a specific connective tissue disorder [6]. It is diagnosed by having a strong anti-ribonucleoprotein (RNP) antibody positivity [6]. Some of the distinct clinical features of MCTD include Raynaud’s phenomenon (RP), swollen digits/puffy hands, arthritis and interstitial lung disease [6]. The cause of MCTD is still unknown but some studies have supported a human leukocyte antigen (HLA)-linked genetic component [6]. Mixed connective tissue disease carries a favorable prognosis and a low-dose corticosteroid requirement [7]. However, current literature questions whether MCTD should truly be considered an individual condition since the diagnostic criteria are vague [7].

Scabies is a common parasitic infestation of the skin caused by *Sarcoptes scabiei,* also known as the itch mite [8]. It has a worldwide distribution but is more prevalent in resource-poor tropical areas [8]. Diagnosis of scabies is primarily made by a patient’s clinical presentation, which may be described as a pruritic rash, worse at night, with a positive history of exposure [8]. The rash is often described as erythematous papules, excoriations, hemorrhagic crusts, vesicles and may even have pustules and impetigo from secondary bacterial infection [9]. A definitive diagnosis is made by retrieving the mite, its eggs, or fecal pellets with identification under a microscope [9]. However, this requires more time, skill and equipment which may not be readily available in all clinical practices, therefore, diagnosis is mostly made on clinical grounds [9]. Scabies treatment is usually with a permethrin 5% cream or lotion for seven to 10 days which kills both the mites and eggs [9]. Both scabies and autoimmune conditions such as SLE cause a rash, therefore, it is important to differentiate between them especially since there have been reported cases of scabies mimicking the presentation of autoimmune cutaneous disorders [10,11].

We report a case of a young African American female who presented with a pruritic rash and was subsequently diagnosed with SLE/Scleroderma overlap syndrome.

## Case presentation

A 20-year old African American female with a recent diagnosis of scabies presented to the emergency department with a pruritic rash on her face, abdomen, hands and scalp for the past month which was progressively worsening within the past two weeks. The patient recently returned from a vacation in Costa Rica one month before her presentation and after returning, started to develop a rash in the various mentioned areas. She initially visited the emergency department three weeks before admission with these symptoms, was diagnosed with scabies, and was discharged with a course of permethrin 5% topical cream; however, her symptoms persisted. The patient also endorsed an unintentional weight loss of 20 lbs within two months associated with fatigue. For one year prior to admission, she noticed that her fingers turned purple when exposed to cold temperatures, and she described tightness in the skin of her hands. 

**Figure 1:**
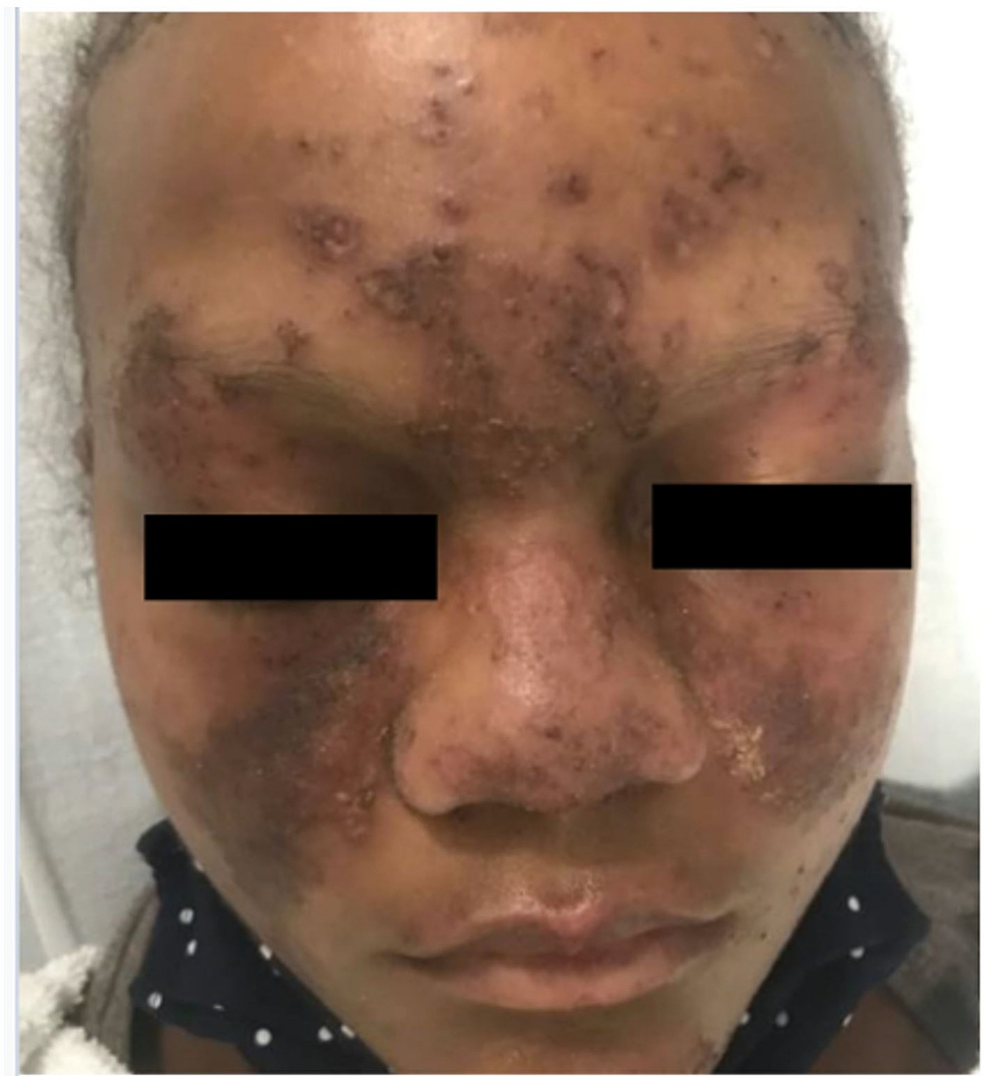
Malar rash with some yellow crusting as well as involvement of the eyelids, forehead and chin

**Figure 2:**
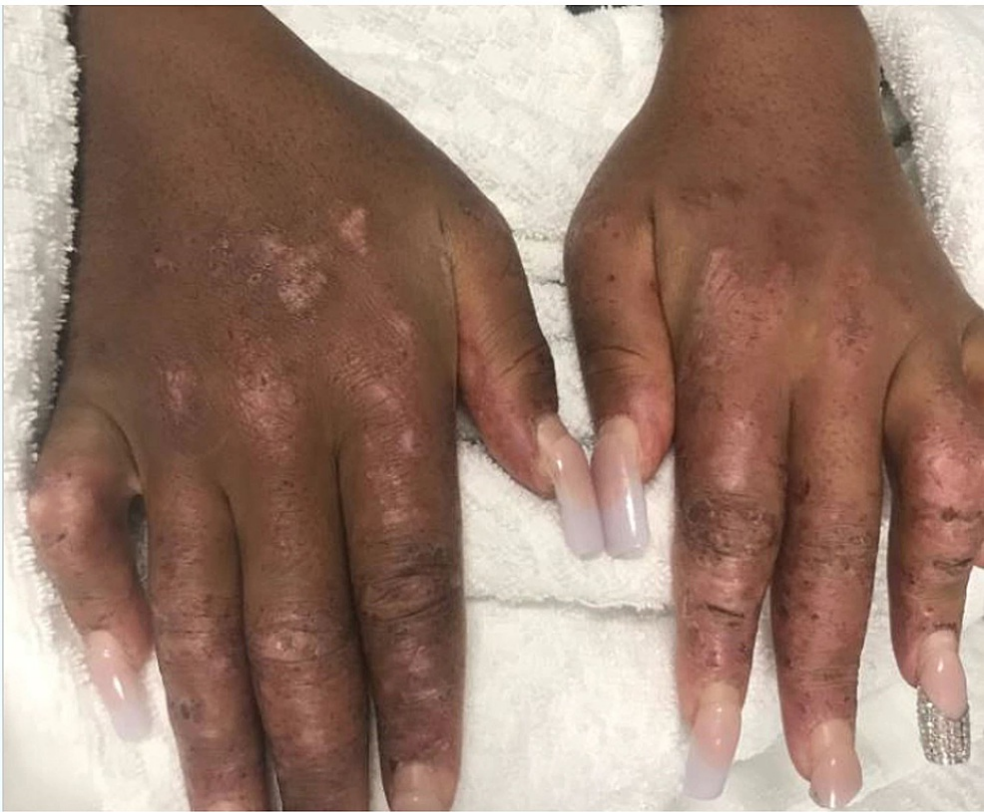
Patchy scaling rash of the hands consistent with a discoid rash

**Figure 3:**
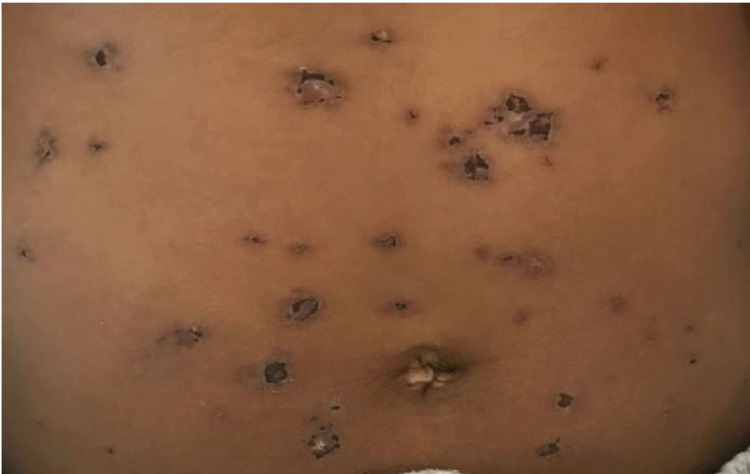
Patchy scaling rash of the abdomen consistent with a discoid rash

**Figure 4:**
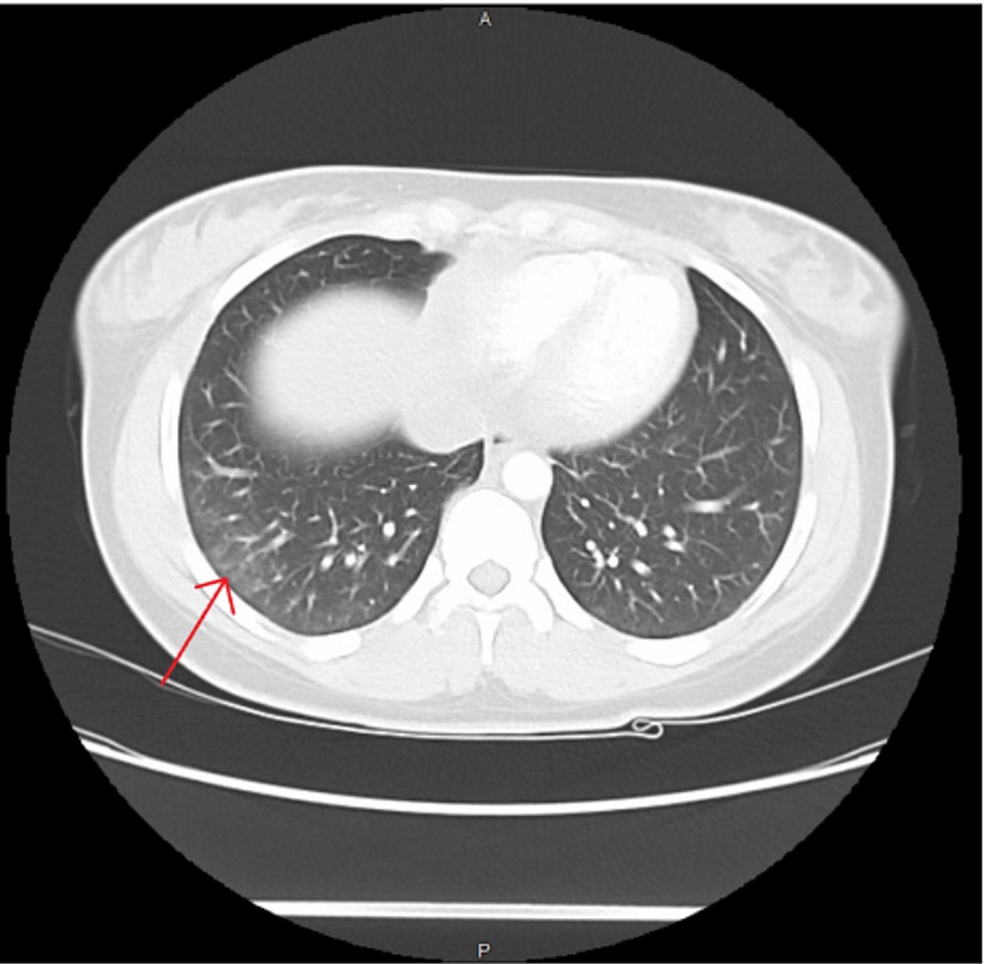
Ground-glass opacities of the right lower lung lobe noted on CT angiogram

On physical examination, she was febrile to 102.2F (39C) and had a pulse of 122 beats per minute. The patient had a weight of 162 lbs (73.5 kg), a height of 65 inches and a body mass index (BMI) of 27.0. A malar rash with yellow crusting was noted on the face, eyelids, forehead, and chin (Figure 1). A patchy scaling rash was also noted on the bilateral hands and the abdomen, consistent with a discoid rash (Figure 2, 3). A buccal aphthous ulcer was noted on the right side of the oral cavity but mucous membranes were moist. Initial complete blood count was significant for pancytopenia with white blood cell count 4000/mcL, hemoglobin 9.8 g/dL, hematocrit 32.2%, mean corpuscular volume (MCV0 93.9 fl, and platelet count 123,000/mcL. Urinalysis was positive for trace ketones, 30 mg/dL protein, 2.0 mg/dL urobilinogen, small leukocyte esterase, 5 to 6 high power field (HPF) white blood cells and 4 to 6 HPF red blood cells. D-dimer was checked in the emergency department and found to be elevated at 1,009 ng/mL. A subsequent CT angiogram was significant for ground-glass opacities in the right lower lung lobe, prominent bilateral axillary lymphadenopathy and a small pericardial effusion with no pulmonary embolism identified (Figure 4). The patient was admitted to the medicine service with the impression of newly diagnosed SLE along with her persistent fever and tachycardia likely secondary to systemic inflammatory response syndrome (SIRS). Initially, she was administered ceftriaxone and azithromycin empirically for possible sepsis, however further work-up was unremarkable for systemic infection. The patient was initially given enoxaparin for venous thromboembolism prophylaxis but was soon changed to rivaroxaban for better compliance.

During the initial course, the patient persistently spiked fevers despite administration of acetaminophen, and would routinely break out in sweats after its administration. The patient’s blood count progressively worsened with her WBC count dropping to a low of 1880/mcL on day five and hemoglobin of 7.6 g/dL (24.5% hematocrit) on day four. The differential diagnoses included autoimmune hemolytic anemia with superimposed anemia of chronic disease likely secondary to an autoimmune process, therefore, low-dose corticosteroid therapy consisting of methylprednisolone 30 mg IV twice daily was started on day four while autoimmune serologies were pending. Rivaroxaban was discontinued due to the risk of bleeding and the patient was put on an intermittent pneumatic compression device (IPCD) for venous thromboembolism prophylaxis. Further infection laboratory work-up was ordered to rule out occult or uncommon viral infections, including varicella, herpes simplex virus (HSV) and dengue virus, in light of the patient’s widespread crusting rash on the body and oral aphthous ulcers, likely representative of a resolving viral exanthem.

By the end of day four, most of the autoimmune serologies were completed and resulted in positive anti-Sjögren's-syndrome-related antigen A autoantibodies (SSA Ab), anti-Smith Ab, anti-RNP Ab, scleroderma-70 (Scl-70) Ab, direct antiglobulin (DAT) poly, DAT C3, beta 2 microglobulin Ab, antinuclear antibody (ANA) titer 1:640 speckled, and borderline ds-DNA Ab (Table 1). The rheumatology team had a concern for hemophagocytic lymphohistiocytosis (HLH) or some other infiltrative disease of the bone marrow due to the patient’s persistent neutropenia despite 24 hours of methylprednisolone treatment and recommended a bone marrow biopsy. A kidney biopsy to further evaluate the proteinuria on urinalysis was considered, however, deferred as the patient retained normal kidney function and the risk of bleeding from the kidney biopsy given the patient's thrombocytopenia outweighed the diagnostic yield. A bone marrow biopsy was also deferred since the ferritin was elevated at 618 ng/mL but not in the range for HLH. A transthoracic echocardiogram (TTE) showed a left ventricular ejection fraction of 55% to 60%, normal right ventricular systolic function, and presence of a small pericardial effusion consistent with the patient’s initial CT angiogram and prominent apical trabeculations. Cardiology was consulted and recommended further evaluation with a nonurgent cardiac MRI as an outpatient.

**Table 1:** Autoimmune laboratory findings during the patient's hospitalization

Autoimmune Marker	Value
anti–Sjögren's-syndrome-related antigen A autoantibodies (SSA Ab)	> 8.0 AI (Positive)
anti–Sjögren's-syndrome-related antigen B autoantibodies (SSB Ab)	< 0.2 AI (Negative)
Anti-Smith Ab	7.2 AI (Positive)
Anti-RNP Ab	7.1 AI (Positive)
Anti-Jo 1 Ab	< 0.2 AI (Negative)
Anti-Scleroderma-70 (Scl-70) Ab	> 8.0 AI (Positive)
Direct antiglobulin test (DAT) Poly	Positive
DAT IgG	Negative
DAT C3	Positive
Beta-2 Microglobulin	3.5 mg/L (Positive)
Cardiolipin Ab	Negative
Antinuclear Ab (ANA) Titer	1:640 (Positive), Homogenous, Speckled Pattern
Rheumatoid Factor (RF)	<10 IU/mL (Negative)
Anti-dsDNA Ab	46 IU/mL (Positive)
Centromere Ab	<0.2 AI (Negative)
Beta 2 Glycoprotein 1 Ab	Negative
Histone Ab	1.0 units (Weak Positive)

**Figure 5:**
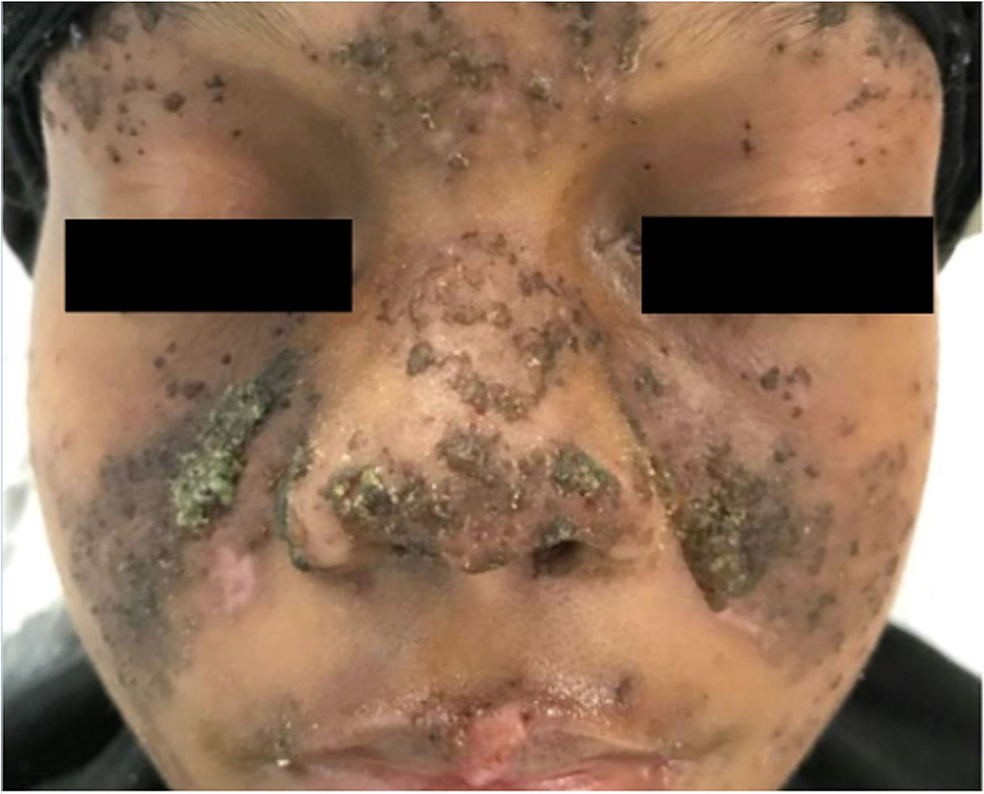
Malar rash crusting over and improving after methylprednisolone treatment

**Figure 6:**
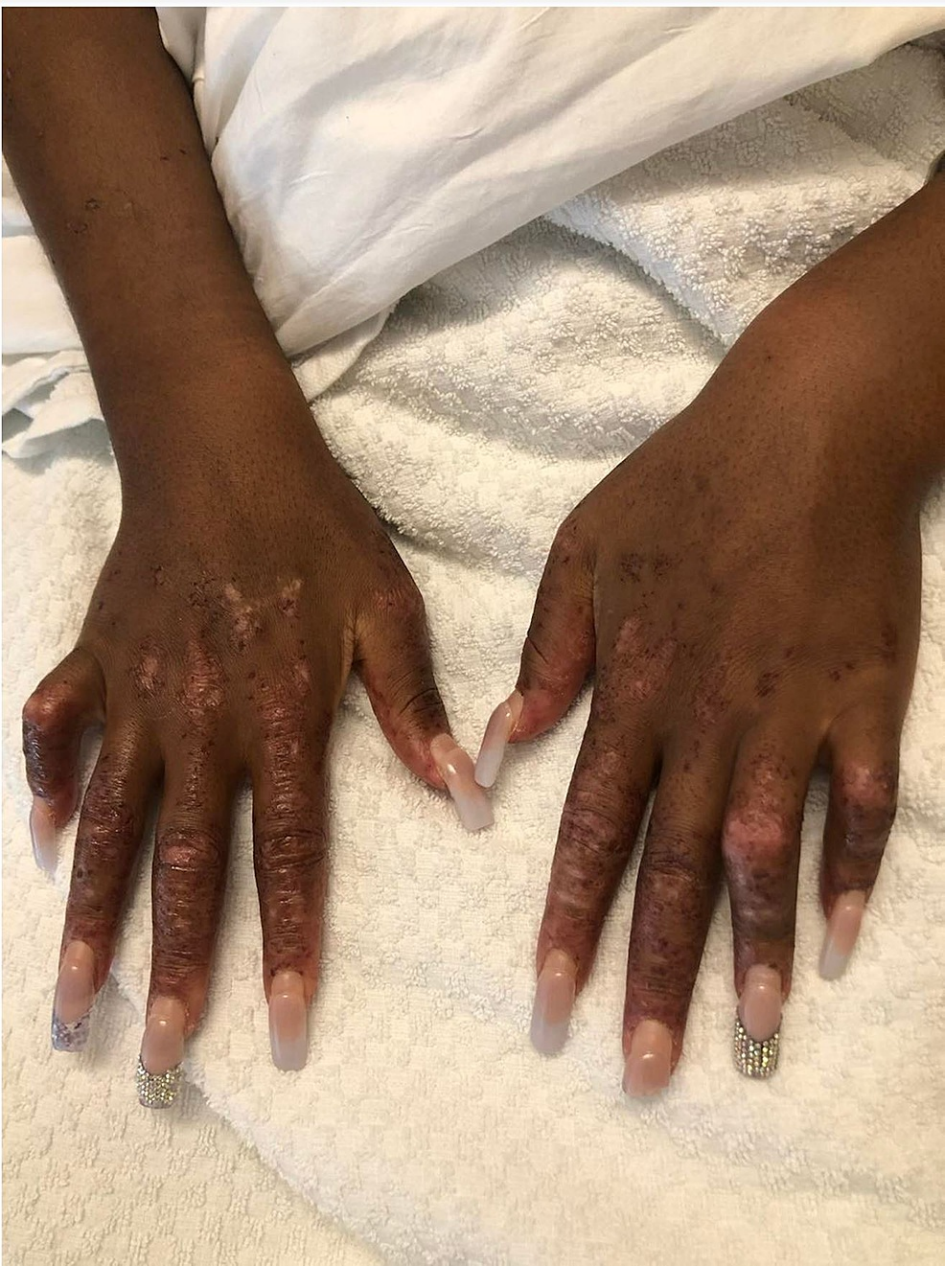
Discoid rash of the hands improving after methylprednisolone treatment

By day eight of hospitalization, the patient was afebrile and her skin rashes were improving with corticosteroids (Figure 5, 6). Upon discharge, the treatment for the patient’s rashes included topical mupirocin 2% ointment to the eroded areas twice daily along with oral doxycycline 100 mg twice daily for one week with patient counselling on the photosensitizing nature of the medication. Polymerase chain reaction (PCR) of the skin lesion swab was negative for varicella and HSV. The discharge diagnosis for this patient was SLE/Scleroderma overlap syndrome since she fulfilled the diagnostic criteria for both connective tissue disorders. The patient was to be followed by multidisciplinary care as an outpatient from rheumatology, hematology/oncology, cardiology, nephrology and dermatology.

## Discussion

Systemic lupus erythematosus is a chronic autoimmune disease with many different clinical presentations [1]. Our patient exhibited cutaneous manifestations in the form of a malar rash and discoid rashes of the hands and abdomen. A biopsy was not obtained of the discoid rashes to confirm the condition, however, clinical presentation supported this diagnosis. Subsequently, she was found to have hematologic involvement (pancytopenia), cardiac involvement (pericardial effusion and prominent trabeculations) and possible renal involvement given the patient’s microscopic hematuria and trace proteinuria [1,2]. She also had a history of Raynaud's phenomenon, ground-glass opacities in the right lower lung lobe (possible interstitial lung disease) and tightening of the skin of the hands, which was likely a sign of sclerodactyly. These features were suggestive of scleroderma [12]. The diagnosis of MCTD would be consistent with a patient that has signs of both SLE and scleroderma along with a positive anti-RNP Ab, however, there are inconsistencies in the criteria required for a diagnosis of MCTD in the current literature [7]. Additionally, the diagnosis of MCTD would only be used when a patient does not fulfil the diagnostic criteria of a single connective tissue disorder but has features of them [6,13]. The term overlap syndrome is preferred when patients fulfil the diagnostic criteria for multiple connective tissue disorders, though some consider MCTD as a type of overlap syndrome [13]. At present, there are four published MCTD criteria all of which include anti-RNP as the immunological marker but anti-RNP antibodies, even at high titer levels, lack specificity for MCTD [7]. In our case, the final diagnosis on discharge was SLE/Scleroderma overlap syndrome since the patient fulfilled the diagnostic criteria for both SLE and scleroderma [2,12,13]. More studies are necessary to ascertain the diagnostic criteria needed for MCTD and assist in the classification of undifferentiated autoimmune rheumatic diseases.

Cases of scabies masquerading as SLE have been described in the current literature, however, it is limited. Bastian et al. presented a similar case of a young Caucasian woman with a pruritic facial rash [10]. The authors found a positive ANA of 1:320 with skin preparation significant for scabies mite infection [10]. Similarly, in a case presented by Werbel et al., a middle-aged African American female with immune thrombocytopenic purpura (ITP), malar erythema, and a desquamative skin rash on the chest and arms was diagnosed with a scabies infection [11]. A skin sample preparation was not obtained in our patient during her initial emergency room visit but, the diagnosis of scabies is mainly made based on the patient’s clinical presentation [8]. In cases of scabies with atypical presentations, the designation of scabies surreptitious has been used [14]. It is, therefore, important to be able to differentiate between the subtle differences of rashes caused by autoimmune etiology such as in SLE vs. an infectious etiology such as in scabies.

## Conclusions

Systemic lupus erythematosus is a well-defined autoimmune disease that can involve many different body systems. When features of other autoimmune conditions are present such as Raynaud’s phenomenon or sclerodactyly along with a positive U1RNP antibody (U1-RNP Ab) test, the term MCTD may be used to describe the patient’s condition since it encompasses features of multiple autoimmune connective tissue diseases but does not fully fulfil the diagnostic criteria of one of them. Overlap syndrome is used to describe patients fulfilling the diagnostic criteria of more than one connective tissue disorder. More research is required to classify patients with MCTD distinctly. Additionally, scabies has been reported to mimic autoimmune disorders that have cutaneous manifestations. Patients with a widespread pruritic rash should still have SLE or other connective tissue disorders on the differential diagnosis, especially if cutaneous manifestations do not resolve with standard therapy. 
